# Evaluation of mandibular anterior alveolus in different skeletal patterns

**DOI:** 10.1186/s40510-016-0135-z

**Published:** 2016-07-20

**Authors:** Nga Hoang, Gerald Nelson, David Hatcher, Snehlata Oberoi

**Affiliations:** Division of Orthodontics, School of Dentistry, University of California, San Francisco, USA; DDI Imaging Center, Sacramento, CA USA; Division of Craniofacial Anomalies, School of Dentistry, University of California, San Francisco, USA; Division of Orthodontics, School of Dentistry, University of California, 707 Parnassus Ave, D-3000, San Francisco, CA 94143 USA

## Abstract

**Background:**

The boundaries for orthodontic tooth movement are set by the bony support of the dentition. This study compares the mandibular anterior alveolar housing in individuals with low, average, and high mandibular plane angles before orthodontic treatment and measures alveolar bone loss and root resorption after orthodontic treatment.

**Methods:**

Pretreatment cone-beam computed tomography (CBCT) images of 75 non-growing individuals, 25 in three groups: low-angle (sella-nasion to mandibular plane ≤28°), average-angle (30°–37°), and high-angle (≥39°), were analyzed. Buccolingual bone thickness was measured at the root apex, mid-root, and alveolar crest of the mandibular right central incisor. Pre- and posttreatment CBCT images of 11 low-angle, 20 average-angle, and 27 high-angle patients were compared to determine changes in the alveolus and mandibular incisor root after orthodontic treatment.

**Results:**

The pretreatment anterior alveolar bone widths were significantly different, wider in low-angle than in average- and high-angle individuals (*p* value = 0.000). High-angle individuals also had greater posttreatment external root resorption, even though the bony housing changed minimally.

**Conclusions:**

Negative sequelae of orthodontic treatment are more frequently found in individuals with high mandibular plane angles and could be linked to their thin pre-existing alveolar housing.

## Background

Biologic factors such as the supporting bone and thickness of the cortical plate as well as biomechanical factors are closely related and determine the potential unwanted side effects of orthodontic treatment, such as external root resorption, gingival recession, dehiscence, and fenestration [1–3]. There is increased prevalence and severity of apical external root resorption in incisors the more the roots are displaced and the longer the treatment [4]. In addition to length of treatment and distance of root displacement, bone boundary conditions can have negative sequelae on orthodontic treatment and thus can limit the ability to provide ideal orthodontic treatment [5].

The dimensions of the mandibular alveolus appear to limit orthodontic tooth movement. Challenging these boundaries may lead to iatrogenic sequelae. Studies have shown that movement of the teeth through cancellous bone causes compensatory remodeling of the bone, while cortical bone does not exhibit the same level of plasticity. Consequently, when the teeth are moved to contact with the cortical plate, movement is slowed. High pressure can force continued movement, but may lead to fenestration, dehiscence of the bone, or root resorption, rather than non-destructive bone remodeling [6].

Several studies have demonstrated a correlation between facial type and alveolar bone morphology of the mandible. In 1996, Handelman et al. showed that vertical growth strongly correlated with alveolar bone thickness, with low mandibular plane angle cases displaying thicker bone lingual to maxillary and mandibular incisors and high mandibular angle cases displaying thinner bone labial to the mandibular incisors [7]. There appeared to be a direct relationship between increased facial and alveolar height and thinness of the alveolar bone, presumably because the incisors continue to erupt to maintain overbite, and the alveolus becomes attenuated with thinning of the width between the labial and lingual walls.

In 2007, Yamada et al., using computed tomography, found that thin alveolar bone anteroposteriorly was associated with high mandibular plane angles and class III malocclusions [8].

More recently, Gracco et al., using computed tomography, confirmed the findings of the previously published two-dimensional studies which showed that the total thickness of the symphysis was greater in the short-face than in the long-face subjects [9] but did not make a connection between thin alveolus and clinical sequelae. In a study on cone-beam computed tomography (CBCT) evaluation of periodontal and bone support loss in extraction cases, the authors concluded that the buccolingual bone thickness was reduced after treatment in both groups, with no differences between the extraction and control groups [10].

It has been reported that CBCT can be used for highly accurate linear quantifications of external apical root resorption [11, 12]. In this retrospective study using CBCT data obtained as part of standard patient records, we evaluate the mandibular anterior alveolus of pretreatment and posttreatment records of adults. We not only describe a correlation between alveolus dimensions and skeletal facial type, but also measure changes to the mandibular alveolus and the lower incisor root length as a consequence of orthodontic treatment.

### Specific aims

1.  Compare the alveolar bone support (height and width) of mandibular central incisor in subjects with low, average, and high mandibular angle skeletal patterns before orthodontic treatment using CBCT.
2.  Measure alveolar bone thickness change and root resorption of mandibular central incisor in the three skeletal patterns following orthodontic treatment.

## Methods

### Aim 1: Evaluation of mandibular anterior alveolar bone before orthodontic treatment

Committee on Human Research (CHR) approval was obtained from the Institutional Committee on Human Research (IRB 10-00564). Pretreatment CBCT images of 75 non-growing individuals, 25 in three groups: low-angle (sella-nasion to mandibular plane (SN-MP) ≤28°), average-angle (30°–37°), and high-angle (≥39°), were analyzed. Consent to use individual CBCT data for research was obtained at the time of taking the CBCT. Buccolingual bone thickness was measured at the root apex, mid-root, and alveolar crest of the mandibular right central incisor. Inclusion criteria were non-growing, no orthodontic treatment before the initial CBCT scan, and no recorded craniofacial anomaly.

**Fig. 1:**
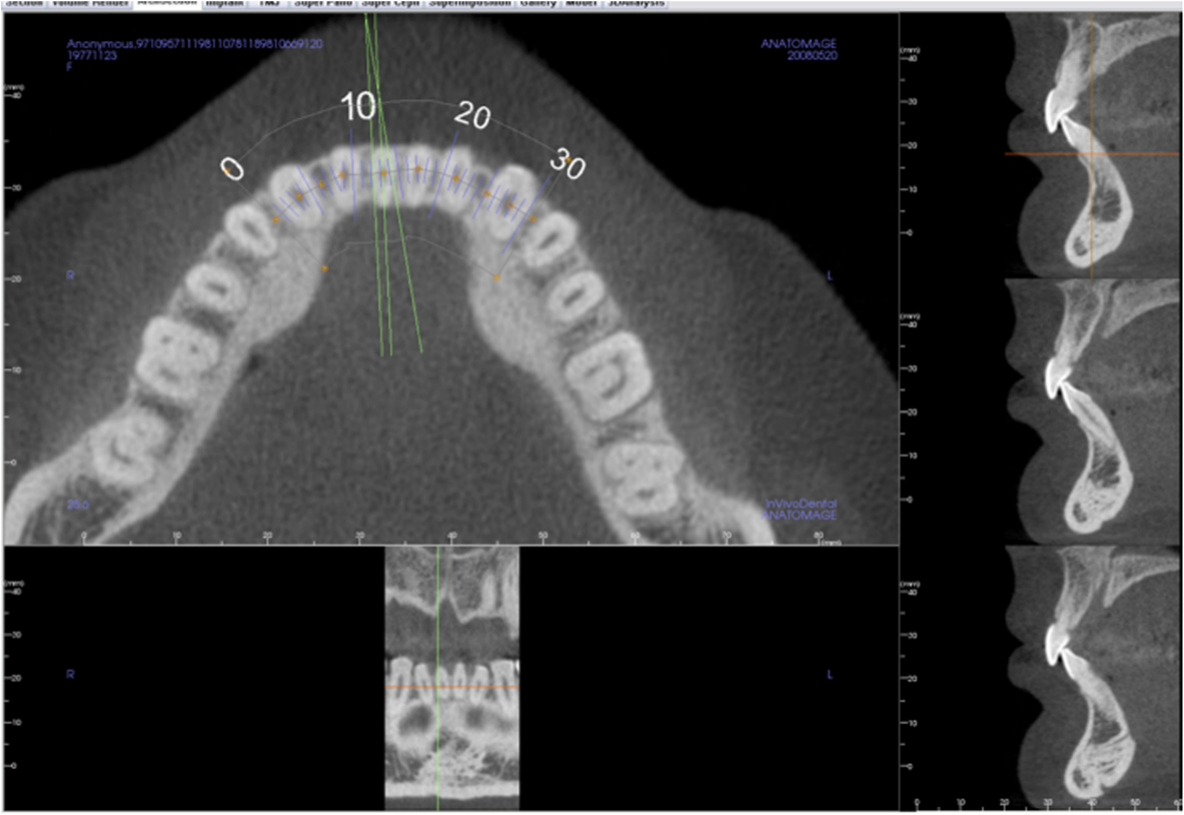
Horizontal view through the mandibular dentition indicating the location of the sagittal slices to evaluate the alveolar bone and teeth. The slice through the center of the root canal (middle slice on the right) is then used for measurement of the tooth and surrounding bony support

**Fig. 2:**
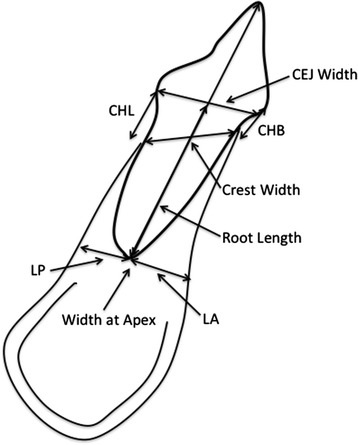
Schematic of measurements taken through the sagittal view of the tooth and mandibular alveolar bone. *CHB* crestal height buccal, measured from the CEJ to the buccal crestal bone, *CHL* crestal height lingual, measured from the CEJ to the lingual crestal bone, *LA* lower anterior bone thickness at the apex, *LP* lower posterior bone thickness at the apex, *crest width* the width of the alveolar bone from the buccal crestal bone to the lingual crestal bone, *CEJ width* the width of the CEJ, *root length* measured from the incisal tip to the apex of the root, *width at apex* width of the alveolar bone at the apex of the tooth, perpendicular to the long axis of the tooth

Using Anatomage Invivo5 CBCT software, sagittal slices were taken of the CBCT image through the middle of the root canal, at the midpoint of the long axis of the right central incisor (Fig. 1). From the sagittal slice of the center of the incisor, pretreatment measurements were taken for the buccolingual width at the apex and the alveolar crest (Fig. 2).

### Reproducibility measurement

To test observer reliability, the measurements were repeated for ten subjects in each group, 1 month apart by the same investigator. The small difference in the measurements at the two observation times indicated reproducibility of the method.

### Statistical analysis

The *F* test, or one-way analysis of variance (ANOVA), was used for statistical analysis. The means and standard deviations of each value were also calculated. When ANOVA was significant at 95 %, the Bonferroni correction was applied to verify where the statistically significant differences were correlated.

### Aim 2: Evaluation of bony and tooth changes after orthodontic treatment

CHR approval (IRB 10-00564) covered this part of the study as well. Fifty-eight subjects seen at the orthodontic clinic between the dates of January 2005 to July 2012, and verified by superimposition of initial and final cephalograms as non-growing, were randomly chosen and categorized into low, average, and high mandibular angle skeletal patterns based on the SN-MP using the same criteria as in the control group described in aim 1.

**Fig. 3:**
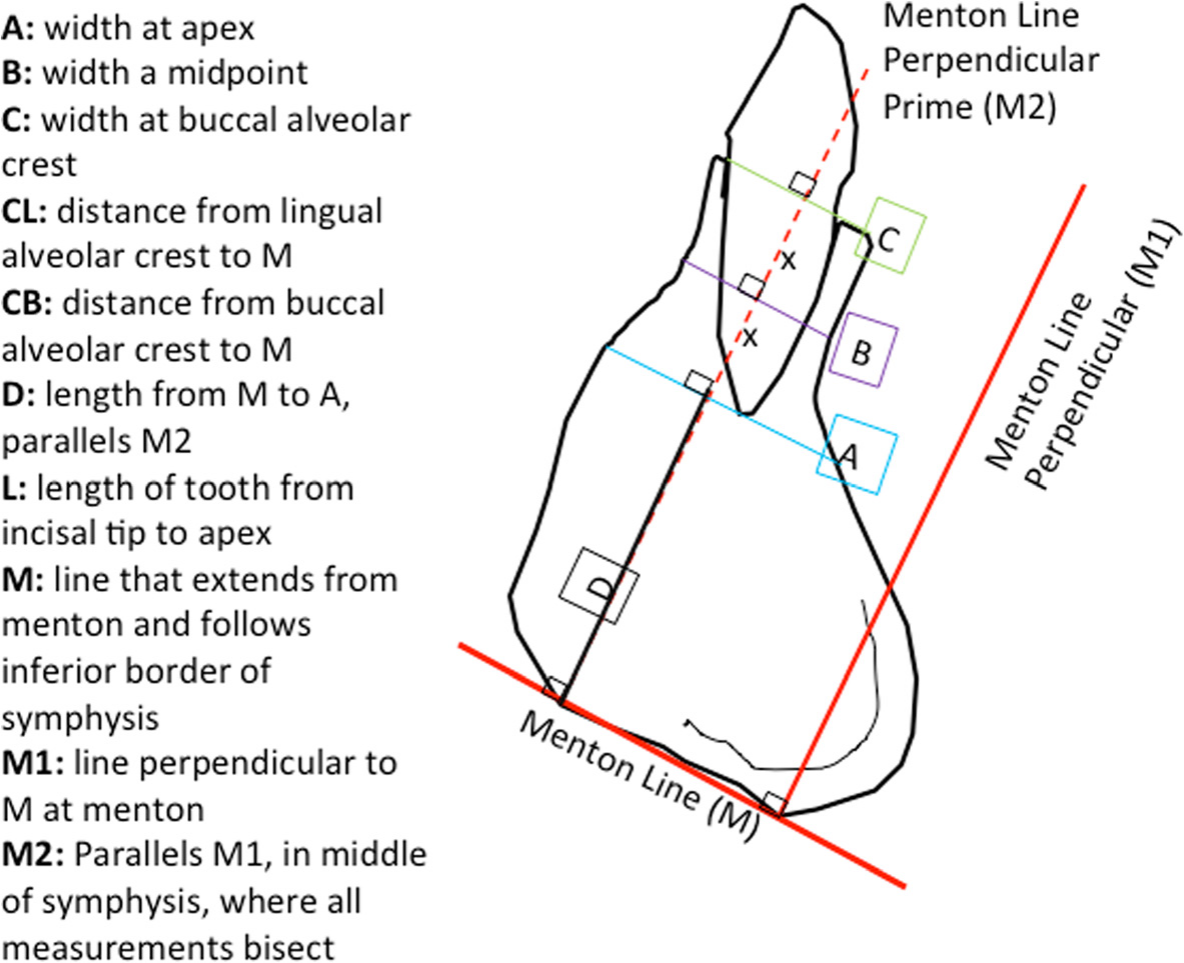
Schematic of measurements determined from a sagittal view of the tooth outlining the width and distances to determine changes in the tooth and alveolar bone

Using the Invivo5, Anatomage software, superimpositions of the pretreatment and posttreatment scans were completed using five landmarks under the Point Registration Module of the software: mental foramen (left and right), nasion, and orbitale (left and right). The two volumetric images were then adjusted by the investigator to make the two images superimpose on the cranial base after using the Point Registration Superimposition Module. The superimpositions were then verified on the axial, sagittal, and coronal planes. The buccolingual bony changes were assessed at the apex, mid-root, and alveolar crest, and the root lengths before and after treatment were compared (Fig. 3) by first evaluating the pretreatment scan and then toggling to the posttreatment scan. It has been reported in the literature that tooth movement will on average cause a 10 % shortening of the roots [13–15]. In this study, any root resorption above 10 % was considered to be out of the norm and, thus, a negative sequela.

### Intra-observer reliability

Thirty CBCT scans (ten from each skeletal pattern group) were measured 1 month apart to test reproducibility of measurements by the same observer. The average difference in the root length measurement between the two time points was 0.254 mm. The average difference in the thickness at the apex between the two time points was 0.21367 mm, and the average difference between the thicknesses from crest to crest was 2.24 mm.

The root length measurement was very reproducible. This reproducibility spans across SN-MP (increase in SN-MP does not change the reproducibility of the measurement), even though the alveolar morphology, as indicated in the findings of aim 1, changes. The alveolar bone thickness at the apex of the lower incisor can also be measured reliably by the same observer at two different time points. The total buccolingual thickness at the alveolar crest was not as reliable as the root length and width at the apex. This is probably due to the difficulty of locating the alveolar crest on the CBCT when the crest is thin.

## Results

### Aim 1: Difference in bony support of mandibular incisors in low-, average-, and high-angle adult subjects with no orthodontic treatment

**Fig. 4:**
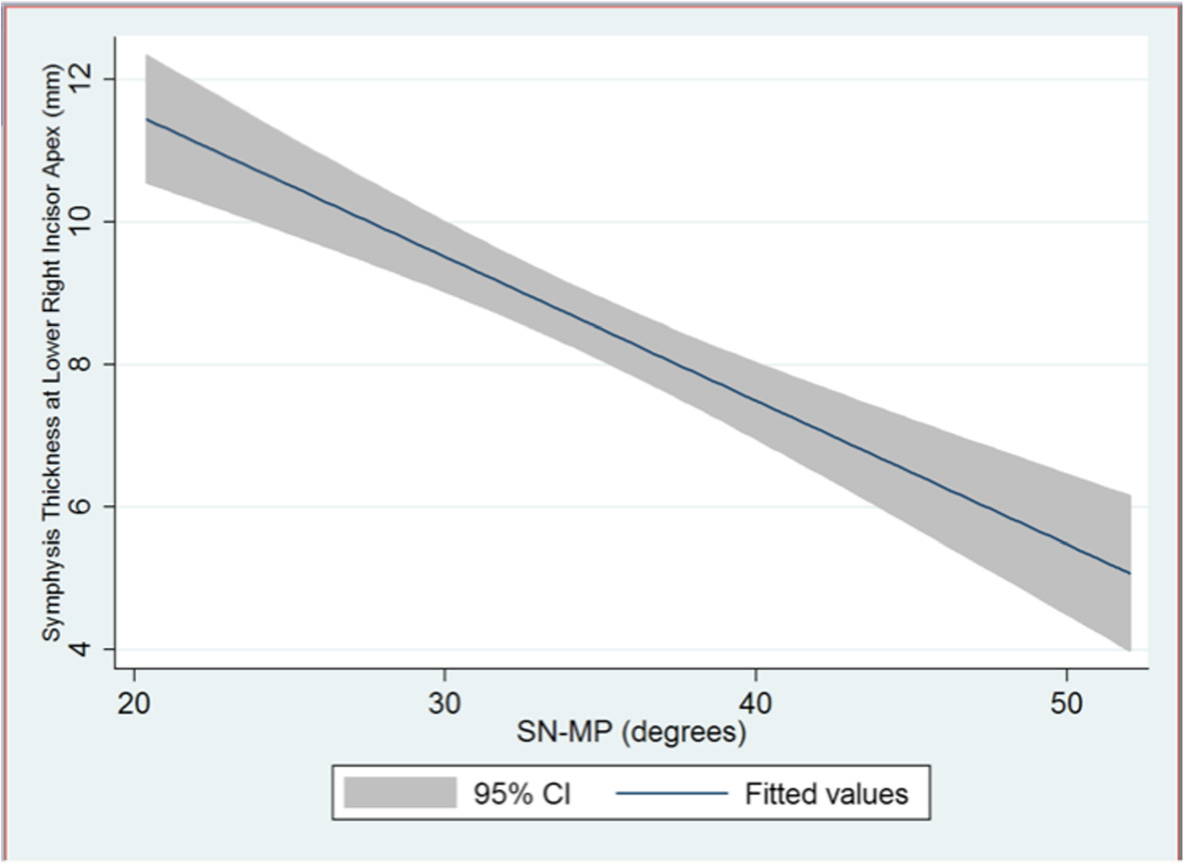
Symphysis thickness at the lower right incisor apex versus SN-MP with 95 % confidence intervals

Our results indicate that there is a relationship between the thickness of the mandibular symphysis at the apex of the lower incisor and mandibular plane angle. As SN-MP increases, the symphysis thickness decreases (*R*^2^ = −0.412, *R* = −0.6418, *p* value = 0.000). Figure 4 shows the decreasing trend clearly with a 95 % confidence interval. We can infer from this trend that two standard deviations (12°) from the average SN-MP of 33°, there is a corresponding 2.42 mm change in the symphysis thickness at the apex of the lower right central incisor.

An ANOVA shows that the difference in the symphysis thickness at the apex in the three groups (low-, average-, and high-angle) is highly statistically significant, with *p* = 0.000. A Bonferroni correction shows that there is a statistically significant difference when comparing the low-angle group with the average- and high-angle groups (*p* = 0.000), but the difference between average- and high-angle groups is not statistically significant (*p* = 0.059).

**Fig. 5:**
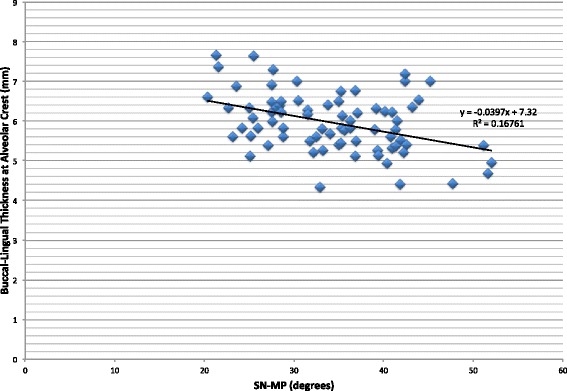
Buccolingual thickness at the alveolar crest of the mandibular right central incisor versus mandibular plane angle. There is a negative correlation between the buccolingual bone thickness at the alveolar crest and SN-MP (*r* = −0.41, *p* value = 0.003)

The difference in the buccolingual thickness at the alveolar crest among the three skeletal patterns is less pronounced than at the apex (Table 1). As seen in Fig. 5, as SN-MP increases, the thickness at the alveolar crest decreases, and though with an *R* value of −0.41, the trend is statistically significant (*p* = 0.003). An ANOVA shows that the differences among the three skeletal groups are significant (*p* = 0.0056), but a Bonferroni correction shows only a statistically significant difference between the low- and high-angle groups (*p* = 0.005).

**Table 1:** Summary statistics of patient population at the mandibular alveolar apex and the alveolar crest

	Number of patients	Mean thickness at the apex (mm)	Std. dev. at the apex	Mean thickness at the alveolar crest (mm)	Std. dev. at the alveolar crest
Low angle	25	10.54	2.06	6.32	0.68
Average angle	25	8.35	1.88	5.88	0.61
High angle	25	7.05	1.80	5.68	0.78

### Aim 2: Differences in bony support and mandibular incisors after orthodontic treatment in low-, average-, and high-angle patients

**Fig. 6:**
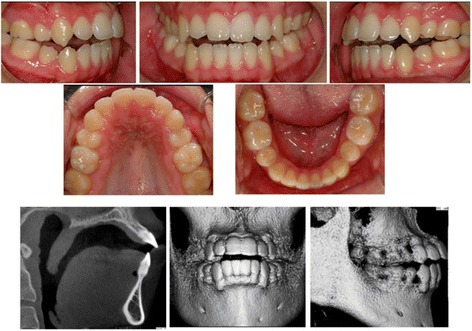
Result of orthodontic treatment in a high-angle patient. No bony issues can be detected clinically, but CBCT images show lower incisors with severe root resorption on a very thin mandibular anterior bony support

**Fig. 7:**
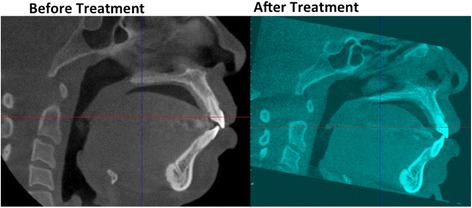
Pretreatment (*black* and *white*) and posttreatment (*blue*) CBCT images of a high-angle patient. Note that the central incisor has been pushed past the buccal cortical plate after orthodontic treatment

There are negative sequelae to orthodontic treatment when the bony support is narrow. Severe root resorption can be seen on CBCT in the mandibular incisors of high-angle cases after orthodontic treatment that cannot be seen clinically (Fig. 6). Our study found that high-angle subjects are more prone to root resorption beyond that expected for routine orthodontic treatment (Fig. 7). Root resorption after orthodontic treatment over 10 % was higher in high-angle compared to average- and low-angle subjects (22, 20, and 9 %, respectively). More alarming was that the high-angle subjects were at the highest risk of having the tooth penetrate the alveolar housing after orthodontic treatment (22 vs. 5 % in average-angle patients and 0 % in low-angle patients) (Table 2). Even though the percentage of individuals with root resorption over 10 % is high in the high-angle patients, Fisher’s exact test yielded a *p* value that was not statistically significant (*p* = 0.824). Fisher’s exact test also showed that the difference in the percentage of the sample population with the teeth penetrating the cortical plates was not statistically significant among the three groups (*p* = 0.138). This could be due to the small sample size of the lower angle patients.

**Table 2:** Root resorption and roots out of the bone after orthodontic treatment in low-, average-, and high-angle patients

	Average initial root length, mm (S.D.)	Number of patients with root resorption over 10 %	Percentage of patients with root resorption over 10 %	Percentage of patients with the tooth out of the bone
Low-angle	20.75 (1.7)	1	1/11 = 9 %	0/11 = 0 %
Average-angle	20 (1.6)	4	4/20 = 20 %	1/20 = 5 %
High-angle	20.5 (0.8)	6	6/27 = 22 %	6/27 = 22 %

Mean bone loss after orthodontic treatment as measured at the apex of the central incisor, mid-root, and alveolar crest ranged between 0.24 and 0.58 mm in all three skeletal patterns (Table 3).

**Table 3:** Summary of mean bone loss at the apex, mid-root, and alveolar crest in low-, average-, and high-angle patients

	Mean bone width loss at apex (S.D.)	Mean bone width loss mid-root (S.D.)	Mean bone width loss alveolar crest (S.D.)
Low-angle	0.24 (0.28) mm	0.54 (0.4) mm	0.30 (0.43) mm
Average-angle	0.25 (0.37) mm	0.20 (0.27) mm	0.28 (0.43) mm
High-angle	0.42 (0.39) mm	0.58 (0.50) mm	0.24 (0.29) mm

An ANOVA of the mean bone loss in the three different skeletal patterns at the apex, mid-root, and alveolar crest showed no significance at the apex and alveolar crest, but there was a significant difference at the mid-root. A Bonferroni correction of the ANOVA that showed a difference at the mid-root only detected a difference between the average- and high-angle patients, with the greater bone loss in the high-angle subjects.

## Discussion

The position of the mandibular incisors is an important factor during orthodontic diagnosis and treatment planning. Studies have shown that prolonged orthodontic treatment is a risk factor for progressive bone loss in individuals whose symphysis is thin and elongated [16].

Lateral cephalometric radiographs are commonly used in conjunction with the clinical exam in evaluating the inclination of the incisors and the thickness of the symphysis. However, radiographic images of the labial and lingual surfaces of the alveolar processes are projected from the more anterior and posterior parts of the bone and do not correspond precisely to the region of the incisors. Also, there is geometric enlargement error from divergence of the X-ray beam [9].

CBCT allows three dimensional assessment of the alveolar support of the incisors without the disadvantages of conventional two-dimensional radiography including image distortion and superimposition. CBCT also allows for secondary reconstructions for qualitative and quantitative of the bone surfaces and quantitative evaluation of the relationship between the teeth and bone [17].

Our study compared the alveolar bone support of mandibular incisors in subjects with different vertical skeletal patterns before orthodontic treatment using CBCT. We found that the mandibular alveolar buccolingual bone thickness at the apex of the lower incisors is larger in the hypodivergent group than in the normodivergent and hyperdivergent groups (*p* value = 0.000). These findings confirm the results shown by Handelman et al. with lateral cephalometric radiography and by Grecco et al. with CBCT [7, 9]. However, the difference between the normo- and hyperdivergent groups was not statistically significant (*p* value = 0.059). When we compared the buccolingual thickness at the apex as a function of increasing SN-MP, we found a negative relationship, with the thickness decreasing with an increase in SN-MP (*R* = −0.64, *p* = 0.000), which is consistent with the findings of previous studies [7–10]. The difference in the buccolingual thickness of the mandibular anterior bone extends above the apex of the root; this difference can be seen even at the level of the alveolar crest, markedly between the low-angle and high-angle subjects (*p* value = 0.005). The difference in the bony support can be attributed to the dentoalveolar compensation that takes place in a hyperdivergent pattern, where the teeth and alveolus hyper-erupt to compensate for the skeletal discrepancy.

The pretreatment morphologic differences in the mandibular anterior alveolar bony support of the lower incisors have some deleterious effects on the teeth after orthodontic treatment. Our study showed that high-angle subjects were at a higher risk of having external root resorption beyond that normally expected for routine orthodontic treatment, with an incidence of 22 % versus 20 % in the average-angle group and 9 % in the low-angle group. Because the pre-existing bony support in the high-angle subjects is so thin, there is a higher risk of moving the incisors out of the cortical bone, either buccally or lingually, during orthodontic treatment, 22 % versus 5 % and 0 % in the average- and low-angle groups, respectively. The differences were not statistically significant, but this could be due to the low sample size of the low-angle group, which was set at SN-MP less than or equal to 28°. It was difficult to find patients to include in our study with such a hypodivergent mandible who did not have orthognathic surgery for correction, and since orthognathic surgery was an exclusion criterion, we could not include them.

The buccolingual thickness decreased minimally after orthodontic treatment (ranged from 0.24 to 0.58 mm) in the three skeletal groups. An ANOVA test showed that the difference in bone loss at the apex and the alveolar crest was not statistically significant but was significant at mid-root. However, a Bonferroni correction of this difference at mid-root only showed a difference between the average- and high-angle subjects, with no difference found when comparing the low and high or average and low groups. This is most likely due to measurement errors, since the differences were small.

## Conclusions

The following conclusions were derived from the comparison of alveolar bone support of the mandibular right central incisor in subjects with different vertical skeletal patterns:

1. Total alveolar buccolingual bone thickness at the apex of the lower right central incisor is larger in the hypodivergent group compared to that in the normodivergent and hyperdivergent.
2. High-angle subjects are more prone to root resorption beyond that expected for routine orthodontic treatment.
3. The amount of buccolingual bone loss in all three skeletal patterns was found to be minimal after orthodontic treatment.

Pretreatment differences in the bony support of the lower incisors in hyperdivergent individuals carry negative consequences associated with orthodontic treatment, with an increase in external root resorption rate and a higher frequency of the tooth penetrating the buccal or lingual cortical plate. CBCT allows us to see these changes that are often masked by superimpositions of structures of the left and right sides in a lateral cephalogram. The clinician should pay careful attention to the pretreatment alveolar bony support of the lower incisors when diagnosing and treatment planning. Special care should be taken in hyperdivergent individuals with a thin symphysis to prevent negative sequelae during orthodontic treatment.
